# Estrogens Induce Expression of Membrane-Associated Estrogen Receptor α Isoforms in Lactotropes

**DOI:** 10.1371/journal.pone.0041299

**Published:** 2012-07-23

**Authors:** Sandra Zárate, Gabriela Jaita, Jimena Ferraris, Guadalupe Eijo, María L. Magri, Daniel Pisera, Adriana Seilicovich

**Affiliations:** Instituto de Investigaciones Biomédicas, Facultad de Medicina, Universidad de Buenos Aires, Buenos Aires, Argentina; Baylor College of Medicine, United States of America

## Abstract

Estrogens are key to anterior pituitary function, stimulating hormone release and controlling cell fate to achieve pituitary dynamic adaptation to changing physiological conditions. In addition to their classical mechanism of action through intracellular estrogen receptors (ERs), estrogens exert rapid actions via cell membrane-localized ERs (mERs). We previously showed that E2 exerts a rapid pro-apoptotic action in anterior pituitary cells, especially in lactotropes and somatotropes, through activation of mERs. In the present study, we examined the involvement of mERα in the rapid pro-apoptotic action of estradiol by TUNEL in primary cultures of anterior pituitary cells from ovariectomized rats using a cell-impermeable E2 conjugate (E2-BSA) and an ERα selective antagonist (MPP dihydrochloride). We studied mERα expression during the estrous cycle and its regulation by gonadal steroids in vivo by flow cytometry. We identified ERα variants in the plasma membrane of anterior pituitary cells during the estrous cycle and studied E2 regulation of these mERα variants in vitro by surface biotinylation and Western Blot. E2-BSA-induced apoptosis was abrogated by MPP in total anterior pituitary cells and lactotropes. In cycling rats, we detected a higher number of lactotropes and a lower number of somatotropes expressing mERα at proestrus than at diestrus. Acute E2 treatment increased the percentage of mERα-expressing lactotropes whereas it decreased the percentage of mERα-expressing somatotropes. We detected three mERα isoforms of 66, 39 and 22 kDa. Expression of mERα66 and mERα39 was higher at proestrus than at diestrus, and short-term E2 incubation increased expression of these two mERα variants. Our results indicate that the rapid apoptotic action exerted by E2 in lactotropes depends on mERα, probably full-length ERα and/or a 39 kDa ERα variant. Expression and activation of mERα variants in lactotropes could be one of the mechanisms through which E2 participates in anterior pituitary cell renewal during the estrous cycle.

## Introduction

Estrogens comprise a group of steroid hormones with a pivotal role in physiological processes. Not only are they essential for female reproductive functions, regulating the hypothalamic-pituitary-ovary axis as well as the normal functioning of the mammary gland, but they also exert numerous actions on non-reproductive tissues such as bone, liver, brain and heart [1].

17β-estradiol (E2) is the prevailing endogenous estrogen in adult females before menopause [2]. It elicits its multiple actions via binding to and activating intracellular estrogen receptors (ER), ERα and ERβ, which function as ligand-dependent transcription factors regulating expression of target genes in the nucleus [3]. However, other mechanisms of E2 action involving rapid activation of membrane-associated ERs and triggering of second-messenger pathways have also been described. Unlike transcriptional events, membrane-initiated events are regulated in short time frames and usually exclude protein synthesis [4]. However, several studies in recent years suggest that membrane-initiated signaling pathways can in turn promote genomic events leading to more long-term consequences [5].

In the anterior pituitary, in addition to well characterized estrogenic actions on gonadotropins and prolactin secretion [6], [7], estrogens are also involved in control of cell fate acting as either a pro-survival, an anti-proliferative or a pro-apoptotic factor [8]–[13]. Estrogens thereby regulate anterior pituitary structural and functional plasticity so that the gland is able to adapt dynamically to changing physiological status and environmental stimuli in several physiological conditions such as pregnancy, lactation and the estrous cycle [14].

Although the anterior pituitary expresses both ERα and ERβ, studies involving knockout mice for either ER isoform have suggested that only ERα plays an important role in this gland [1]. ERα protein is found primarily in lactotropes, followed by somatotropes and, to a lower extent, gonadotropes and thyrotropes [15]. Expression of ERβ in the pituitary is lower than that of ERα and remains constant along the estrous cycle [15], [16]. On the contrary, the expression of ERα varies along the estrous cycle, reaching minimum levels at diestrus and maximum at proestrus, which can be positively correlated with E2 circulating levels [15]. Apart from full-length ERα and ERβ, other variants of these receptors have been described in the adult rat pituitary. Early studies in lactotrope-somatotrope-enriched fractions showed the presence of a minor ER isoform of ∼65 kDa, which could correspond to full-length ERα, and two major ER variants of ∼50 and ∼37 kDa. However, the ∼65 kDa variant is the single ER form found in the gonadotrope-enriched population [17]. Whether these variants are produced by alternate ER mRNA splicing or specific posttranslational processing in different cell types remains unknown. Also, the anterior pituitary from the adult female rat expresses a truncated ERα form of ∼20–22 kDa (TERP-1) which is transcribed from a unique promoter and contains part of the hormone-binding domain and C-terminal region of the full-length receptor [1]. TERP-1 protein, localized mainly in lactotropes and gonadotropes [16], can modulate the action of full-length ERs in a positive or negative manner depending on cell and promoter context [1].

The identity of membrane-associated ERs has been quite controversial, but increasing evidence shows that intracellular and membrane ERs are the same proteins. Both ERα and ERβ localize in the plasma membrane as well as other extra-nuclear sites of many cell types [18]. However, truncated forms of ERα, G-protein coupled receptors and other non-related proteins have also been involved in membrane-initiated estrogen signaling [19]–[32]. A 46 kDa amino-terminal truncated product of full-length ERα with potent inhibition of ERα transactivation activity has been described in the plasma membrane of osteoblasts [19], [20]. In human endothelial cells, this membrane truncated form of ERα was reported to modulate membrane-initiated estrogen actions, including eNOS activation, more efficiently than full-length ERα [21]. In the plasma membrane of hypothalamic neurons and astrocytes, in addition to full-length ERα, a ∼50 kDa variant of this receptor has been identified [22]–[24]. Truncated ERα variants of approximately 52 and 46 kDa have also been described in the membrane of breast cancer MCF-7 cells [25], [26]. Moreover, a novel, predominantly membrane-based 36 kDa variant of ERα has been found in human breast carcinomas and in different breast and endometrial cancer cell lines [27], [28]. Whereas it is clear that gonadal steroids regulate the expression of intracellular ERs [1], it is still debatable whether these hormones regulate ER expression in the plasma membrane.

Trafficking is a dynamic process of re-location of proteins from one region of a cell to another. In hormone-responsive tissues, surface expressed ERs are often in dynamic equilibrium with intracellular receptor pools, so that rapid changes in surface populations can be mediated. These rapid changes in receptor populations are fundamental for the functioning of cells that need to respond quickly and accurately to a changing hormonal environment [14].

We previously showed that E2 exerts a rapid pro-apoptotic action in anterior pituitary cells, especially in lactotrope and somatotrope populations, through activation of membrane-associated ERs [13]. Considering that E2 triggers a proapoptotic signal at the plasma membrane level in anterior pituitary cells and that the nature of the receptors involved in this apoptotic action remains elusive, in this study we investigated the participation of membrane-associated ERα (mERα) in the pro-apoptotic action of E2 in this gland. We and others recently described the presence of mERα in anterior pituitary cells [11], [13]. Now, we evaluated mERα expression during the estrous cycle and its regulation by gonadal steroids in vivo in total anterior pituitary cells, lactotropes and somatotropes, the anterior pituitary cell populations with the highest turnover. We also identified ERα variants in the plasma membrane of anterior pituitary cells during the estrous cycle and studied E2 regulation of these mERα variants in vitro by surface biotinylation.

## Materials and Methods

### Ethics Statement

All animal work was conducted according to the NIH guidelines and was approved by the Institutional Ethical Committee (Protocol # Res. (CD) 2869/10) at the University of Buenos Aires School of Medicine.

### Drugs

All drugs and reagents were obtained from Sigma Chemical Co., St. Louis, MO, USA except for phenol red free Dulbecco’s Modified Eagle Medium (D-MEM) and supplements (Gibco, Invitrogen, Carlsbad, CA, USA), fetal bovine serum (Natocor, Córdoba, Argentina), all terminal deoxynucleotidyltransferase-mediated deoxyuridine triphosphate nick end labeling (TUNEL) reagents (Roche Molecular Biochemicals, Mannheim, Germany), primary antibodies against anterior pituitary hormones (Dr. A. Parlow, National Hormone and Pituitary Program, Torrance, CA, USA), anti-guinea pig rhodamine-conjugated secondary antibody, anti-guinea pig fluorescein isothiocyanate (FITC)-conjugated secondary antibody, streptavidin horseradish peroxidase (HRP) conjugated anti-rabbit antibody (Chemicon International, Temecula, CA, USA) and materials indicated below.

### Animals

Adult female Wistar rats (200–250 g) were kept in controlled conditions of light (12 h light-dark cycles) and temperature (20–25°C). Rats were fed standard lab chow and water *ad libitum*. Rats were ovariectomized (OVX) 2 wk before the experiments under ketamine (100 mg/kg, i.p.) and xylazine (10 mg/kg, i.p.) anesthesia. Cycling rats were monitored by daily vaginal smears. Rats with three or more normal consecutive 4–5 day estrous cycles were killed between 7∶30 am and 9 am of diestrus I or proestrus. For steroid treatments *in vivo*, OVX rats were injected for two consecutive days with vehicle (polyethyleneglycol, VEH), E2 (20 µg/100 g b.w.), P4 (2 mg/100 g b.w.) or a combination of both steroids and killed 24 h after the last injection. Anterior pituitary glands were removed within minutes after decapitation and processed for the experiments below.

### Cell Culture

A pool of anterior pituitary cells from 2–12 OVX rats was used for each culture. Anterior pituitary glands were washed several times with Dulbecco’s Modified Eagle’s Medium supplemented with 10 µl/ml MEM amino acids, 2 mM glutamine, 5.6 µg/ml amphotericin B, 100 U/ml penicillin, 100 µg/ml streptomicin (DMEM-S) and 3 mg/ml bovine serum albumin (BSA). Then, glands were cut into small fragments. Sliced fragments were dispersed enzymatically by successive incubations in DMEM-S-BSA, containing 0.75% trypsin, 10% fetal bovine serum (FBS) previously treated with 0.025% dextran-0.25% charcoal (FBS-DCC) to remove steroids and 45 U/µl deoxyribonuclease type I (Invitrogen, CA, USA). Finally, cells were dispersed by extrusion through a Pasteur pipette in Krebs buffer without Ca^2+^ and Mg^2+^. Dispersed cells were washed and resuspended in DMEM-S with 10% FBS-DCC. Cell viability assessed by trypan blue exclusion was over 90%. Dispersed cells were seeded onto coverslides in 24-well tissue culture plates (1.5×10^5^ cells/ml/well) for the TUNEL assay or onto 24-well tissue culture plates (1−1.5×10^6^ cells/ml/well) for cell surface biotinylation analysis. The cells were cultured for 24 h in DMEM-S with 10% FBS-DCC, then incubated for 0–120 min in phenol red free, serum free DMEM-S supplemented with 0.1% BSA (DMEM-S-BSA 0.1%) containing 1 nM 17β-estradiol (E2), 1 nM 17β-estradiol 6-(O-carboxymethyl)oxime:BSA (E2-BSA, 33 mol E2/mol BSA) or ethanol 1 µl/l. In some experiments, cells were pre-incubated with 100 nM 1,3-Bis(4-hydroxyphenyl)-4-methyl-5-[4-(2-piperidinylethoxy)phenol]-1H-pyrazole dihydrochloride (MPP, Tocris, Ellisville, MO, USA) for 30 min and then co-incubated with MPP and E2-BSA for 120 min.

### Preparation of E2-BSA Devoid of Free E2

Free E2 was carefully removed from E2-BSA solutions before each experiment as previously described [13]. Briefly, an aliquot of 400 µl of E2-BSA (100 µM in E2 dissolved in 50 mM Tris buffer, pH 8.5) was applied to a centrifugal filter unit with a MW cut-off of 3000 (Millipore, Bedford, MA, USA) and centrifuged at 14000×g for 55 min. The retentate was washed three times with the same buffer, recovered and the volume adjusted to 400 µl.

### Microscopic Determination of DNA Fragmentation by TUNEL

After the incubation period, anterior pituitary cells were fixed with 4% formaldehyde in PBS for 10 min and permeabilized by microwave irradiation. DNA strand breaks were labeled with digoxigenin-deoxyuridine triphosphate using terminal deoxynucleotidyl transferase (0.18 U/µl) according to the manufacturer’s protocol. After incubation with 10% normal donkey serum and 10% normal sheep serum in PBS for 40 min, cells were incubated for 1 h with guinea pig rat prolactin (PRL) antiserum (1∶1500) or guinea pig rat growth hormone (GH) antiserum (1∶2000). Then, slides were incubated with fluorescein-conjugated antidigoxigenin antibody (1∶10) to detect incorporation of nucleotides into the 3′-OH end of damaged DNA and rhodamine-conjugated antiguinea pig secondary antibody (1∶200) in the same buffer. Slides were mounted with mounting medium for fluorescence (Vectashield, Vector Laboratories Inc., Burlingame, CA) containing 4′,6 diamidino-2-phenylindoledihydrocloride (DAPI) for DNA staining and visualized in a fluorescent light microscope (Axiophot, Carl Zeiss, Jena, Germany). The percentage of apoptotic anterior pituitary cells was calculated as [(TUNEL+)/total cells] ×100, the percentage of apoptotic lactotropes as [(TUNEL+PRL+)/total PRL+] ×100 and the percentage of apoptotic somatotropes as [(TUNEL+GH+)/total GH+] ×100.

### Localization of Membrane Estrogen Receptor α (mERα) in Anterior Pituitary Cells, Lactotropes and Somatotropes

Anterior pituitary cells from cycling female rats killed at diestrus I or proestrus or from OVX rats treated with VEH, E2, P4 or E2+P4 were dispersed as described above for cell cultures. Dispersed cells were washed, resuspended in PBS-BSA 0.1% and separated into tubes at a density of 4×10^5^ cells/tube. Then, cells were incubated for 1 h at 37°C to stabilize cell membranes [13]. Next, cells were fixed with 0.1% paraformaldehyde in PBS for 10 min at room temperature (RT) in the dark and permeabilized by 10-minute incubation with PBS-saponin 0.05% (MP Biomedicals Inc., OH, USA). Cells were incubated for 1 h with guinea pig rat PRL antiserum (1∶2000) or guinea pig rat GH antiserum (1∶2000) in PBS-saponin 0.05% followed by 40-minute incubation with a FITC-conjugated antiguinea pig secondary antibody (1∶75) in the same buffer. Since cell permeabilization with saponin is reversible [33], cells were washed, resuspended in PBS-BSA 0.1% and rocked for 1 h at 37°C to allow repair of cell membranes. Then, cells were incubated for 1 h with rabbit anti-rat ERα antibody MC-20 (3 µg/10^6^ cells, Santa Cruz Biotechnology, Santa Cruz, CA, USA) in PBS followed by 40-minute incubation with a phycoerithrin (PE)-conjugated anti-rabbit secondary antibody (1∶67, Vector Laboratories Inc., Burlingame, CA, USA) in the same buffer. For isotype controls, cells were incubated with guinea pig serum instead of PRL or GH antiserum and rabbit immunoglobulin instead of ERα antibody. Cells were washed, resuspended in PBS and analyzed by flow cytometry using a FACScan (Becton Dickinson). There was no difference in the percentage of mERα-positive cells between anterior pituitary cells fixed and labeled and those that went through the entire process of fixation, permeabilization, repair and labeling (data not shown). Data were analyzed with WinMDI 98 software.

### Cell Surface Biotinylation

Anterior pituitary cells from cycling female rats killed at diestrus I or proestrus or from OVX rats were cultured in DMEM-S with 10% FBS-DCC for 24 h as described above. After this attachment period, cells from OVX rats were incubated with E2 for 0–120 min as described above. Then, cells from either cycling rats or OVX rats incubated with E2 were processed for cell surface biotinylation [22]–[24], [34]. Approximately 3−4.5×10^6^ cells were used for each condition. The entire procedure was performed at 4°C to prevent exo- and endocytosis. Cells were rinsed twice with ice-cold PBS supplemented with 1.5 mM MgCl2, 0.2 mM CaCl2, pH 7.8 (PBS^2+^), followed by 20-minute incubation with freshly prepared ice-cold PBS^2+^-sulfo-NHS-SS-biotin (2 mg/ml, Pierce Biotechnology Inc., Rockford, IL, USA) with gentle shaking. Excess biotin reagent was quenched by rinsing cells three times with ice-cold quenching solution (100 mM glycine in PBS^2+^) and further incubating them twice in the same solution for 15 min. Cells were lysed by 20-minute vigorous shaking in lysis buffer containing 150 mM NaCl, 1% Igepal, 0.02% sodium azide, 0.1% sodium dodecyl sulphate (SDS) and a protease inhibitor cocktail (1∶50, Sigma) in 50 mM Tris-HCl pH 7.4. Cell lysate was collected and clarified by centrifugation at 16,000×g for 30 min and the supernatant was incubated with Immobilized NeutrAvidin Gel (Pierce Biotechnology Inc.) overnight at 4°C on a tube rotator and spun at 16,000×g for 5 min to collect beads. This supernatant was considered the intracellular fraction. Beads were washed three times with PBS^2+^ containing protease inhibitor cocktail (1∶100). Bound proteins (membrane fraction) were eluted with SDS-PAGE sample buffer supplemented with 100 mM dithiothreitol (DTT) and protease inhibitor cocktail (1∶50) for 30 min at RT on a tube rotator. Eluted proteins were separated from NeutrAvidin beads by centrifugation. Samples were kept at −70°C until analyzed by Western blot.

### Western Blotting

Protein concentration of intracellular fractions was determined by the Bradford protein assay (BioRad Laboratories, CA, USA). 10–15 µg of intracellular proteins or 20 µl of membrane proteins were size-fractionated in 15% SDS-polyacrylamide gel, then electrotransferred to polyvinyl difluoride (PVDF) membranes. Blots were incubated for 90 min in 5% nonfat dry milk-TBS-0.1% Tween 20 at RT and incubated overnight with rabbit anti-rat ERα antibody MC-20 in the same buffer at 4°C. This was followed by 1 hour-incubation with HRP-conjugated anti-rabbit antibody. Immunoreactivity was detected by enhanced chemiluminescence (Productos Bio-Lógicos, Buenos Aires, Argentina). Chemiluminescence was detected by chemoluminiscence imaging system (G Box Chemi HR16, Syngene, Cambridge, United Kingdom) and bands were quantified using Gene Tools software (Syngene). To assess equal loading of gels, intensity data from intracellular proteins were normalized with respect to the corresponding β-actin blot while intensity data from membrane proteins were normalized with respect to corresponding data from reversible Ponceau staining [35]. Data were expressed as relative increment versus respective controls.

### Statistical Analysis

The number of apoptotic cells identified by TUNEL was analyzed in slides from at least three independent experiments. Total anterior pituitary cell number in each slide was evaluated by DAPI nuclear staining. Results were expressed as the percentage of apoptotic cells ±95% confidence limits (CL) of the total number of cells counted in each specific condition. Confidence intervals for proportions were analyzed by χ^2^ test. The percentage of mERα-positive cells (obtained by FACS) was expressed as mean ± SE and evaluated by Student’s *t* test or two-way ANOVA followed by Tukey’s test. Normalized Western blot data were analyzed by paired Student’s *t* test or repeated measures one-way ANOVA followed by Dunnett’s multiple comparisons test. Differences were considered significant if *p*<0.05. All experiments were performed at least three times.

## Results

### Effect of ERα Blockade on E2-BSA-induced Apoptosis of Anterior Pituitary Cells

**Figure 1:**
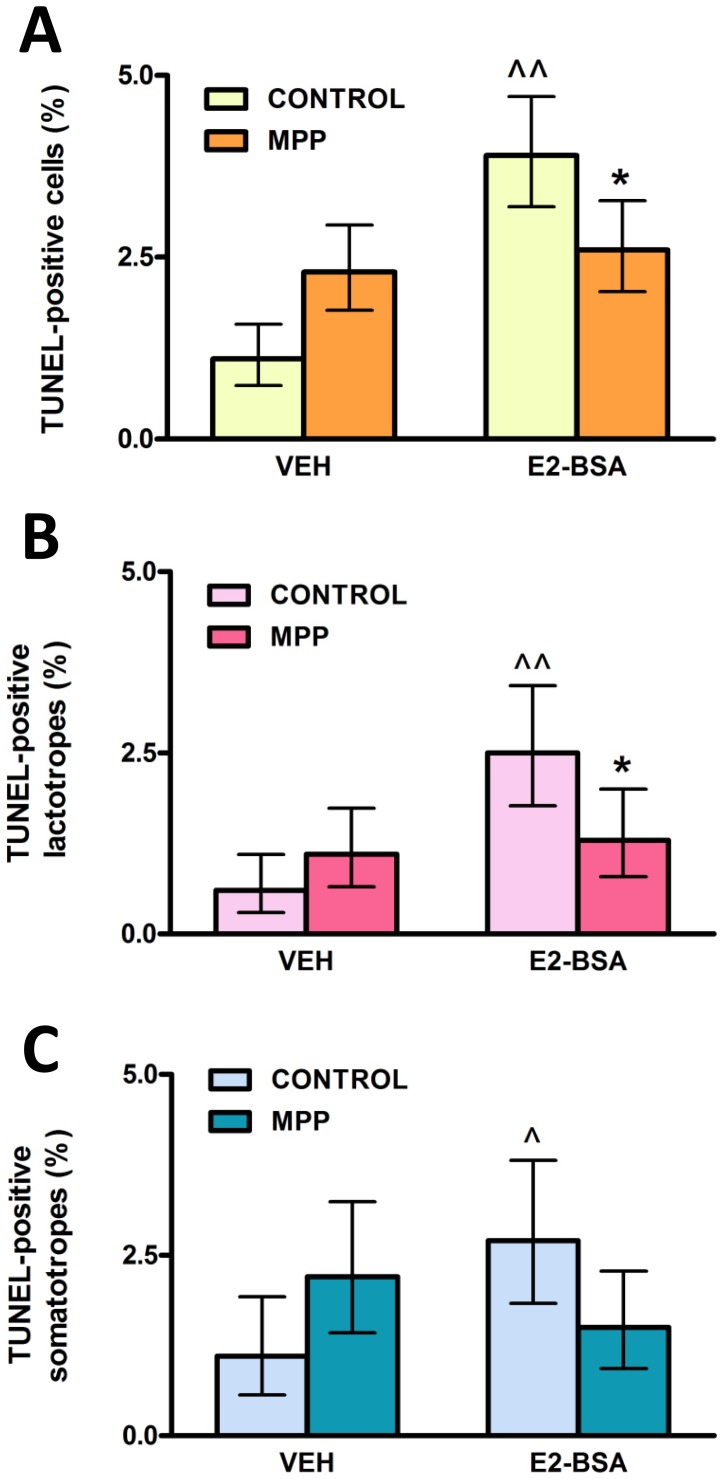
An ERα-antagonist (MPP) blocks E2-BSA-induced apoptosis of lactotropes. Anterior pituitary cells in culture were pre-incubated with MPP (100 nM) for 30 min prior to the addition of E2-BSA (1 nM) or VEH for 120 min. Apoptosis was analyzed by TUNEL and lactotropes and somatotropes were identified by immunocytochemistry. Each column represents the percentage ± CI (95%) of TUNEL-positive cells (n≥2600) (A), lactotropes (n≥1500 cells/group) (B) and somatotropes (n≥1100 cells/group) (C). *p<0.05 vs respective control without MPP; ∧p<0.05, 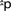
<0.01 vs respective control without E2-BSA, χ^2^ test.

To determine the involvement of ERα in apoptosis of anterior pituitary cells induced by E2 we first studied the effect of MPP, an ERα-selective antagonist, on the apoptotic action triggered at the level of the plasma membrane by membrane-impermeant E2-BSA. E2-BSA (1 nM) increased the percentage of TUNEL-positive anterior pituitary cells after 120 min of incubation and MPP (100 nM) blocked this estrogenic action (Fig. 1 A). Then we ran indirect immunofluorescence assays for PRL and GH to identify TUNEL-positive cells within lactotrope and somatotrope populations. While MPP completely reversed E2-BSA-induced apoptotic action in lactotropes, it non-significantly reversed this estrogenic action in somatotropes (Fig. 1 B, C).

### Expression of Membrane ERα in Anterior Pituitary Cells, Lactotropes and Somatotropes During the Estrous Cycle

**Figure 2:**
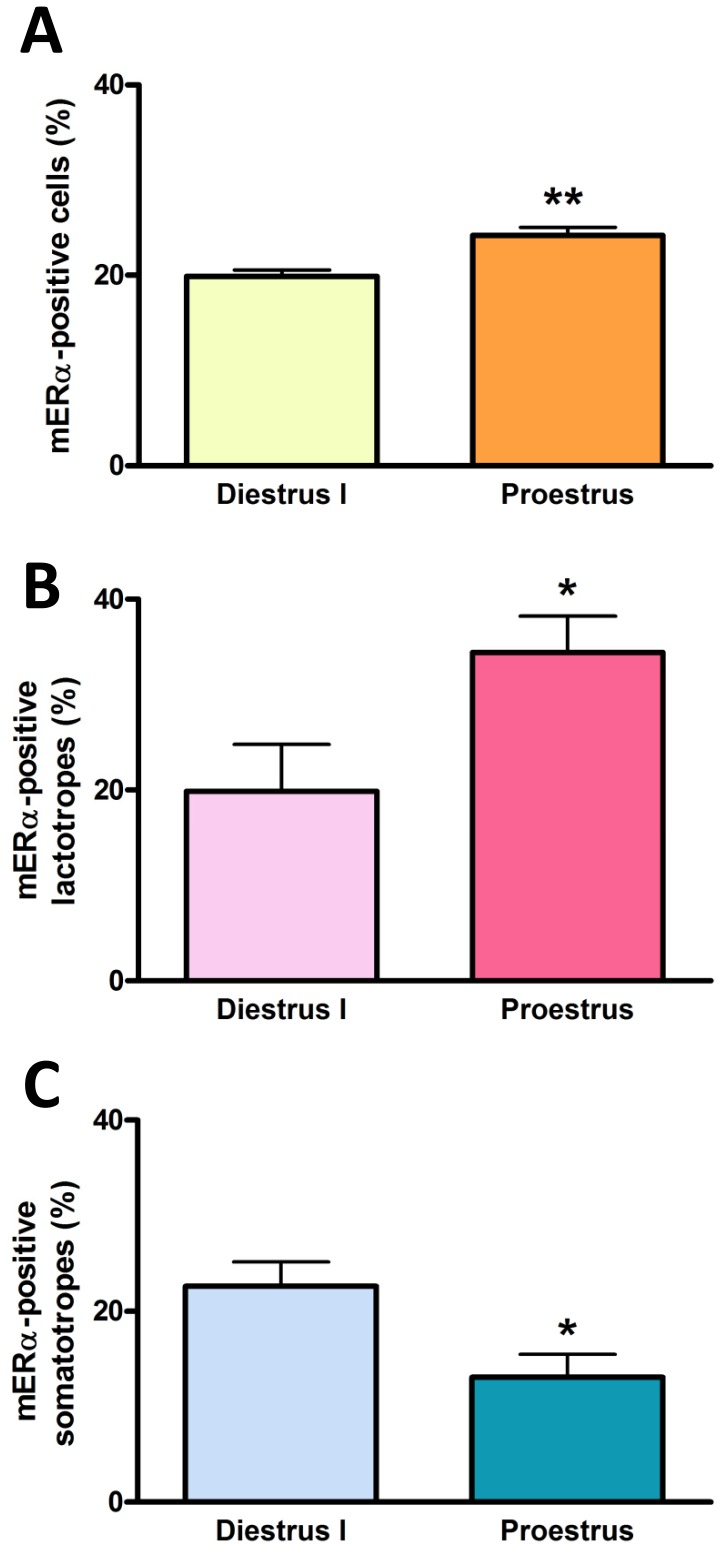
The percentage of lactotropes and somatotropes expressing mERα varies during the estrous cycle. Dispersed anterior pituitary cells from rats euthanized at diestrus I or proestrus were immunostained for both mERα and PRL or GH and analyzed by flow cytometry. Each column represents the mean ± SE of anterior pituitary cells (A), lactotropes (B) or somatotropes (C) expressing mERα (n = 3–4 animals per group). *p<0.05, **p<0.01 vs diestrus I, Student’s *t* test.

In a previous study, we reported the presence of a membrane form of the classical ERα (mERα) in anterior pituitary cells, lactotropes and somatotropes from adult female rats [13]. To explore whether the expression of these membrane receptors varies during the estrous cycle, we ran double indirect immunofluorescence assays for both mERα and either PRL or GH in anterior pituitary cells from cycling rats killed at proestrus or diestrus I. Using an approach that preserves the integrity of the cell membrane and thus excludes the labeling of nuclear receptors [13], we detected a higher percentage of anterior pituitary cells and lactotropes expressing mERα at proestrous than at diestrus I (Fig. 2 A, B). In contrast, the percentage of somatotropes expressing this membrane receptor was lower at proestrus than at diestrus I (Fig. 2 C).

### Effect of Gonadal Steroids on the Expression of Membrane ERα in Anterior Pituitary Cells, Lactotropes and Somatotropes

**Figure 3:**
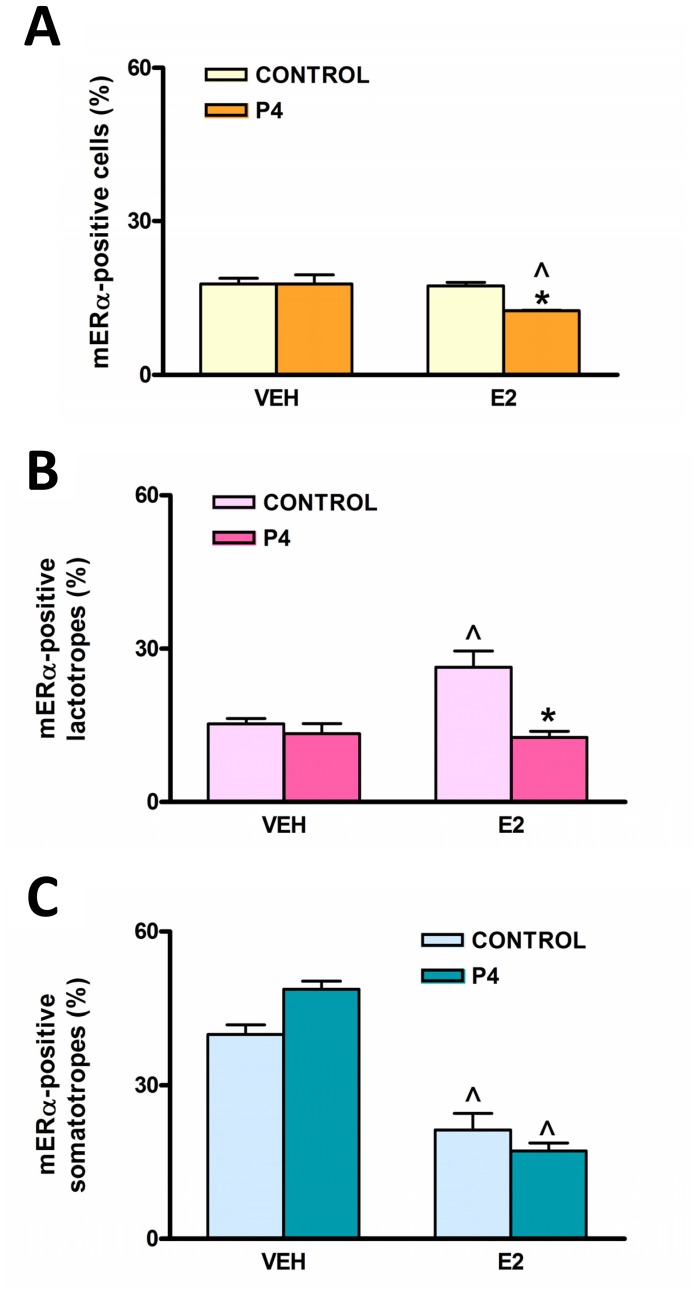
Gonadal steroids differentially regulate the expression of mERα in lactotropes and somatotropes *in vivo*. OVX female rats were injected for two consecutive days with vehicle (VEH), 17β-estradiol (E2), progesterone (P4) or E2+P4 and euthanized on the third day. Dispersed anterior pituitary cells were immunostained for both mERα and PRL or GH and analyzed by flow cytometry. Each column represents the mean ± SE of anterior pituitary cells (A), lactotropes (B) or somatotropes (C) expressing mERα (n = 3−4 animals per group). *p<0.05 vs respective control without P4; ∧p<0.05 vs respective control without E2, two-way ANOVA followed by Tukey’s test.

To study whether gonadal steroids estradiol and progesterone regulate the expression of mERα in the pituitary, we performed the same analysis described above in OVX rats acutely treated with E2, progesterone (P4), or E2 plus P4. In total anterior pituitary cells, neither E2 nor P4 alone had any effect on mERα expression. However, we observed a significant decrease in the percentage of mERα-expressing cells in the group treated with both steroids together (Fig. 3 A). We then analyzed gonadal steroid action on mERα in pituitary populations. Treatment with E2 increased the percentage of lactotropes expressing mERα whereas P4 abrogated this estrogenic effect (Fig. 3 B). Again, somatotropes behaved differently from lactotropes. A high percentage of somatotropes expressed mERα basally and E2 treatment decreased this percentage (Fig. 3 C). P4 treatment, either alone or in combination with E2, did not modify the percentage of somatotropes expressing mERα.

### Identification of Membrane ERα Isoforms in the Anterior Pituitary

**Figure 4:**
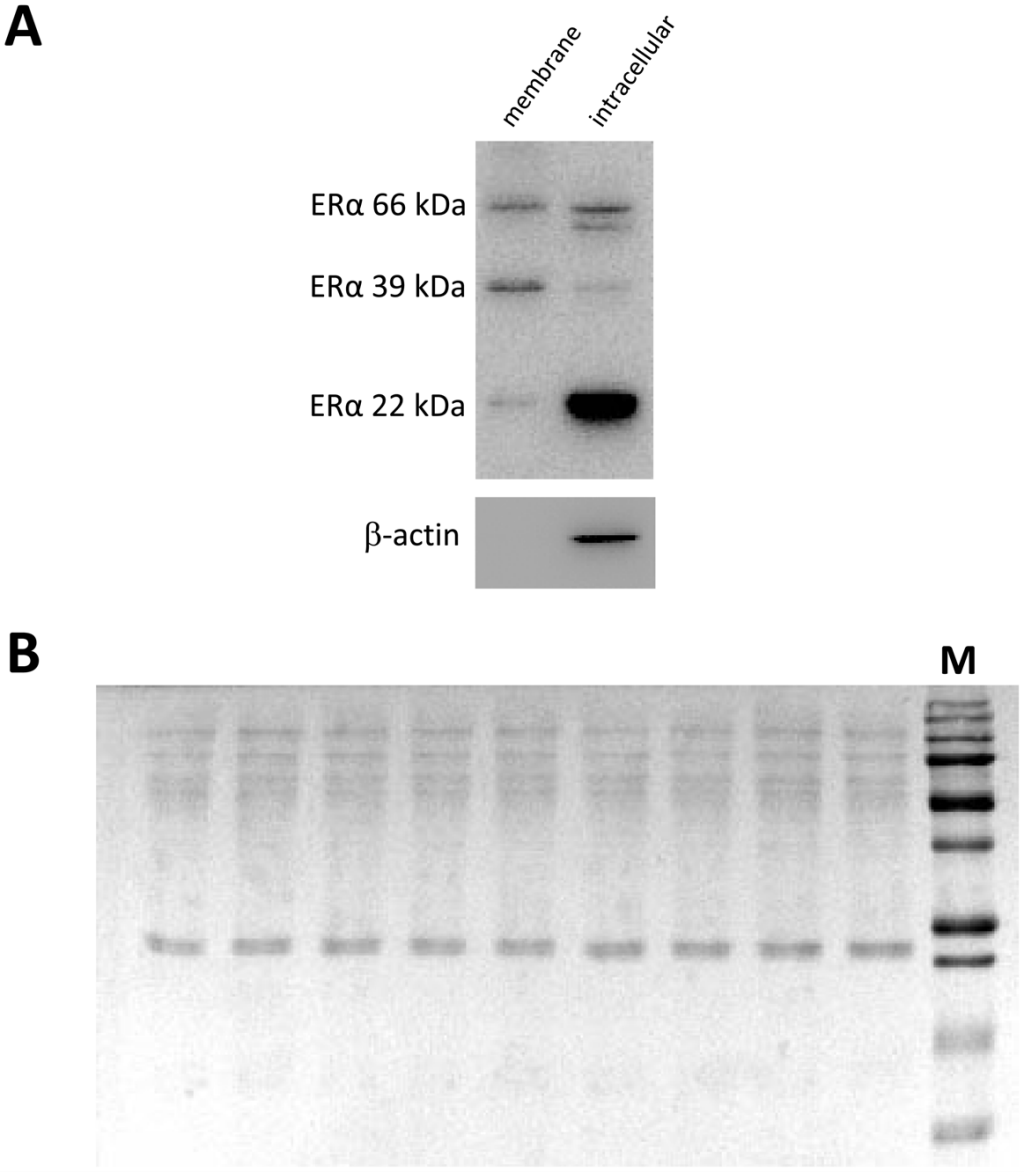
Cell surface biotinylation of anterior pituitary cells. Cultured anterior pituitary cells from cycling rats were processed for cell surface biotinylation as described in *Material and Methods.* Western blots from biotinylated (membrane) and non-biotinylated (intracellular) protein fractions were probed with anti-rat ERα and β-actin antibodies (A). PVDF membranes from gels loaded with membrane protein fractions were stained with reversible Ponceau S to ensure equal loading of proteins (B). M: MW marker.

A number of mERα variants have been recently described in different tissues, where they participate in membrane-initiated estrogen signaling [21], [22], [27], [28]. To characterize mERα isoforms expressed in the anterior pituitary gland, we surface biotinylated anterior pituitary cells from cycling rats with membrane impermeable sulfo-NHS-SS-biotin. Both biotinylated and intracellular fractions showed three ERα-immunoreactive bands of approximately 66, 39 and 22 kDa by Western blot (Fig. 4 A). To ensure that the labeled protein fraction was uncontaminated with intracellular proteins, we probed biotinylated and intracellular fractions against β-actin. Immunostaining for the intracellular marker protein β-actin was negative, thereby verifying the purity of the biotinylated sample (Fig. 4 A). Stained PVDF membranes with reversible Ponceau S showed that biotinylated samples were equally loaded onto SDS-PAGE gels (Fig. 4 B).

### Expression of Membrane ERα Isoforms in Anterior Pituitary Cells During the Estrous Cycle

**Figure 5:**
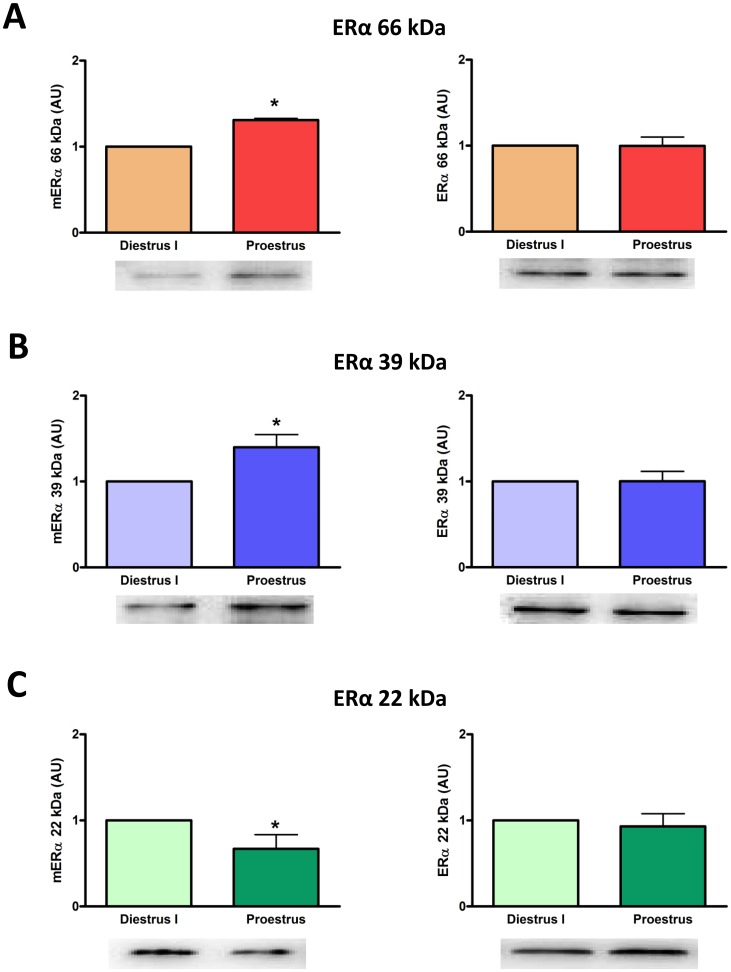
The expression of mERα isoforms varies during the estrous cycle. Anterior pituitary cells from cycling female rats killed at diestrus I or proestrus were cultured for 24 h to allow attachment to the culture plate and then processed for cell surface biotinylation. Expression of full-length ERα (A) and its 39 kDa (B) and 22 kDa (C) isoforms was evaluated by Western blot in membrane (*left panels*) and intracellular (*right panels*) protein fractions. Densitometric data from 3–6 animals per group were normalized by the corresponding Ponceau staining (*left panels*) or β-actin value (*right panels*) and analyzed by paired Student’s *t* test, *p<0.05 vs diestrus I. Each column represents the mean ± SE of the relative increment of proestrus versus corresponding diestrus I.

We examined the expression of mERα isoforms in anterior pituitary cells from rats killed at proestrus or diestrus I. Expression of mERα variants of 66 and 39 kDa was higher whereas the expression of the mERα variant of 22 kDa was lower at proestrus compared to diestrus I (Fig. 5 A, B, C *left panels*). Expression of the three intracellular ERα isoforms remained unchanged between these stages of the estrous cycle (Fig. 5 A, B, C *right panels*).

### Effect of 17β-estradiol on Expression of Membrane ERα Isoforms in Anterior Pituitary Cells

**Figure 6:**
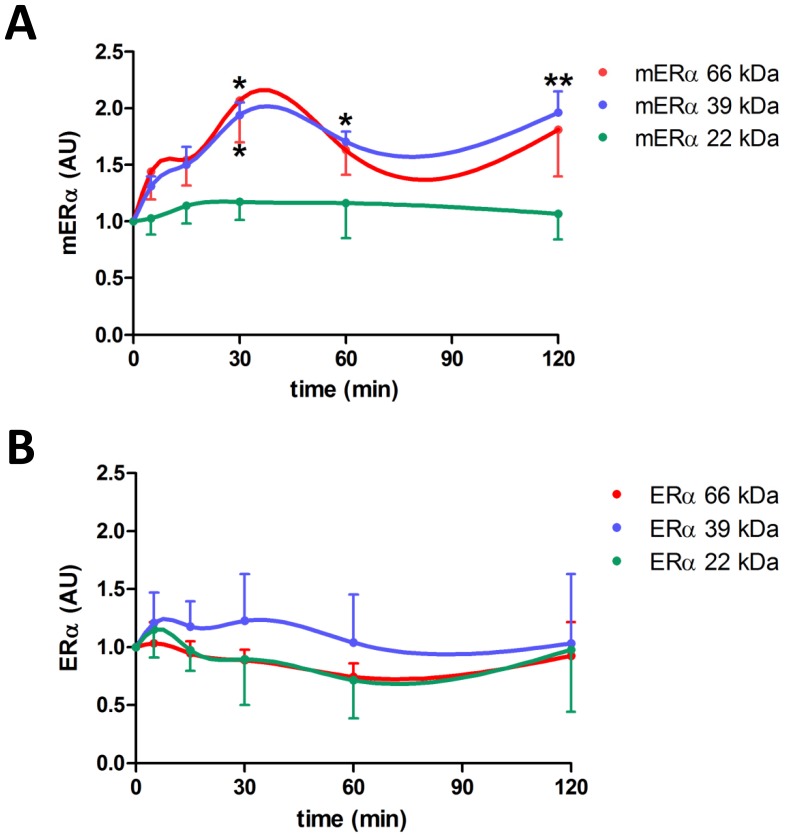
17β-estradiol increases expression of 66 kDa (full-length) and 39 kDa mERα isoforms in a time-dependent manner. Anterior pituitary cells from OVX rats in culture were incubated with E2 (1 nM) for 0–120 min and then processed for cell surface biotinylation. Expression of full-length ERα and its isoforms was evaluated by Western Blot in membrane (A) and intracellular (B) protein fractions. Densitometric data from 3–5 experiments were normalized by the corresponding Ponceau staining (A) or β-actin value (B) and analyzed by repeated measures one-way ANOVA followed by Dunnett’s multiple comparisons test, *p<0.05, **p<0.01 vs 0 min. Each point represents the mean ± SE of the relative increment of each time compared to corresponding time 0 min for each ERα isoform.

We then studied how E2 affected the expression of mERα isoforms in anterior pituitary cells from OVX rats incubated for 0–120 min with this steroid. The cells basally expressed the same three isoforms of 66, 39 and 22 kDa (Fig. 6 A, time 0 min). E2 (1 nM) rapidly increased the expression of 66 and 39 kDa ERα proteins in the plasma membrane. Both isoforms reached significantly different levels after 30 min of E2 incubation. Whereas the increase in the expression of the 66 kDa mERα isoform was transient, levels of the 39 kDa mERα isoform remained elevated for the remaining time points of the experiment (Fig. 6 A). Again, expression of the three intracellular ERα isoforms remained unchanged throughout the experiment (Fig. 6 B). There was no change in the expression of either membrane or intracellular ERα isoforms with long-term (12–24 h) E2 treatment (data not shown).

## Discussion

We previously reported that E2 exerts a rapid apoptotic action in anterior pituitary cells, lactotropes and somatotropes via activation of membrane-associated ERs [13]. We also reported that ICI 182,780, a pure antagonist of both ERα and ERβ, abrogated the estrogenic apoptotic action, suggesting that receptors involved are likely to be classical ERs in a membrane location or other closely related receptor [13]. In the present study, we show that the increase in TUNEL-positive lactotropes in response to a membrane-impermeable E2 conjugate is blocked by MPP dihydrochloride, a selective, high affinity ERα antagonist [36], suggesting that membrane-associated ERα is the likely candidate for mediating E2 rapid induction of apoptosis in this cell population. E2-BSA also induced apoptosis of somatotropes, as previously reported [13]. Although the presence of the antagonist lowered the number of apoptotic somatotropes induced by E2-BSA to control levels, this action was not statistically significant. It has been shown that only a subset of GH-bearing cells responds to gonadal steroid deprivation [37], suggesting the existence of different subpopulations of GH-expressing cells with differential sensitivity to ER activation.

Estradiol is a critical factor in the regulation of several physiological processes in the anterior pituitary gland and, as more studies emerge, it is becoming increasingly apparent that membrane-associated ERs are involved in many of these estrogenic actions. Rapid estrogenic actions via mER leading to hormone release and (anti)proliferative responses have been described in both normal and transformed anterior pituitary cells [11], [38]–[41]. Also, E2 activation of the GnRH receptor gene was recently reported to involve mER in ovine pituitary cells [42]. Most studies indirectly infer the involvement of mERα in these estrogenic actions, either because of high expression of this receptor in pituitary cell lines or absence of ERβ in the plasma membrane of normal pituitary cells [11], [38]–[41]. In the present study, by combining a cell impermeable analog of E2 and a highly selective ERα antagonist, we showed direct participation of mERα in an estrogenic biological effect in normal anterior pituitary cells.

It has been proposed that the expression of mER is controlled more dynamically than its nuclear counterpart [43]. Thus, the presence of mERs in anterior pituitary cells, especially in lactotropes, would allow them to change their sensitivity to estrogens depending on circulating hormonal levels, as occurs along the estrous cycle. In fact, our results show that the number of total anterior pituitary cells and lactotropes expressing mERα was higher at proestrus than at diestrus I. This finding tallies with descriptions of total ERα in the anterior pituitary along the estrous cycle [15]. Interestingly, these authors reported that in almost all stages of the estrous cycle, including the morning of proestrus, ERα immunostaining was predominantly cytoplasmic, particularly localized in rough endoplasmic reticulum membranes and secretory vesicles. However, at midday proestrus, ERα immunoreactivity was located only in the nucleus [15]. Thus, ERα seems to be associated with the endomembrane system when E2 levels are low. As E2 levels start to increase in the early hours of proestrus, ERα-containing vesicles may fuse with the cell membrane and deliver this receptor to the cell surface to mediate membrane-initiated E2 signaling in the anterior pituitary. This mechanism for E2-induced sorting of ERα-containing vesicles to the plasma membrane was reported in hippocampal neurons [44]. The pattern of mERα expression in anterior pituitary cells during the estrous cycle could result from changes in circulating levels of gonadal steroids, since acute E2 treatment to OVX rats increased the percentage of lactotropes expressing mERα. The highest rate of apoptosis in the anterior pituitary is observed in the morning of proestrus and may depend on high E2 circulating levels at this stage of the estrous cycle [45], [46]. Increased apoptosis induced by E2 via mERα could depend, at least in part, on the increase in the number of lactotropes expressing mERα at proestrus. On the other hand, a high percentage of somatotropes from OVX rats expressed mERα, and acute administration of E2 decreased the percentage of mERα-bearing somatotropes. Also, the percentage of somatotropes expressing mERα is lower at proestrus compared to diestrus I. Contrary to the idea of an aimless collection of dispersed cells throughout the anterior pituitary, GH-secreting cells are organized into a spatially restricted three dimensional network that undergoes marked remodeling upon physiological demand in response to central or peripheral signals [47]. The proportion of responsive GH cells within the network was reported to be sensitive to the prevailing gonadal steroid environment, suggesting dynamic regulation of the GH cell network response by gonadal steroids in both male and female mice [48]. Moreover, the existence of substantial homotypic and heterotypic interactions within and between each pituitary lineage network may be involved in exchange of information to ensure balance in relative cell number and/or function between different cell lineages [47]. Although lack of substantial transdifferentiation between somatotropes and lactotropes has been reported in physiological conditions in mice [49], other studies suggest that this may be not the case in rats, where conversion of somatotropes to lactotropes has been suggested to participate in the expansion of this population during lactation [6]. Therefore, the reduction in the percentage of GH-bearing cells expressing mERα both at proestrus and with acute E2 treatment could result from transdifferentiation processes between somatotropes and lactotropes or even other pituitary cell populations. In fact, the cyclic rise in gonadotropin levels during the estrous cycle can be positively correlated with the emergence of a subset of multihormonal somatotropes sharing phenotypic features of gonadotropes. This multihormonal population has been suggested to expand in response to estrogens to support the proestrus surge of gonadotropins [50], [51].

We also observed that progesterone abrogated E2-induced increase in the number of lactotropes expressing mERα whereas it had no effect on E2-induced decrease in the number of mERα-bearing somatotropes. Many actions of estrogens are antagonized by progesterone in the anterior pituitary [52], [53]. This hormone counteracts the stimulatory effect of estrogens on cell proliferation and prolactin release [54], [55]. It also impairs the permissive effect of estradiol on anterior pituitary cell apoptosis induced by TNF-α and FasL [56], [57]. The peak in circulating levels of progesterone that occurs in the afternoon of proestrus may decrease mERα expression in total anterior pituitary cells and lactotropes, thus limiting membrane-initiated estradiol signaling and favoring nuclear-initiated E2 signaling [15].

In addition to full-length ERα, with a molecular weight predicted by its respective coding region, tissue-specific splice variants exist for this receptor [58]. Since ERα variants can differ in intracellular localization and may act alone or may influence full-length ERα activity [1], it is critical to define the natural expression of these ERα forms in physiological conditions. In the pituitary, an estrogen binding protein of ∼65 kDa corresponding to full-length ERα, has been described in both gonadotrope- and lactotrope-somatotrope-enriched total protein fractions in male rats [17]. Also, lactotrope-somatotrope enriched fractions express two additional estrogen binding variants of ∼50 and ∼37 kDa [17]. To date, the functional significance of these lower molecular weight ER proteins is unknown. A truncated form of ERα of ∼20–22 kDa (TERP-1) was also described in the anterior pituitary of female rats in physiological conditions [1]. There is a great bulk of evidence showing that mERs and intracellular ERs are products of the same genes [59]. However, the precise nature of mERs - e. g. full-length or other variants - in different tissues and physiological conditions remains elusive. Surface biotinylation is a tool for labeling membrane proteins exhibiting an extracellular domain [34]. In a recent report, Gutierrez et al [60] only identified the presence of full-length ERα in the plasma membrane of anterior pituitary cells and suggested that it is the major mERα protein. In the present study, we detected the presence of full-length 66 kDa ERα as well as two other variants with lower molecular weights (∼39 kDa and ∼22 kDa) both in cytoplasm plus nucleus and in membrane fractions of anterior pituitary cells. The detection of mERα variants by surface biotinylation implies the presence of an extracellular domain in these receptors. Several arguments account for the existence of an extracellular domain in mERα. In the pituitary tumor cell line GH3, a variety of antibodies recognize different epitopes of ERα under experimental conditions in which cell membrane integrity is preserved [43]. Also, cell-impermeable analogues of E2, such as E2-BSA and E2-peroxidase, induce a rapid increase in intracellular calcium levels and prolactin release in both normal and tumor anterior pituitary cells [11], [38], [61] Prolactin release is also stimulated by ERα-specific antibodies which are too large to enter the cell, indicating the presence of an active extracellular domain in the mERα able to transduce estrogenic signals from the plasma membrane [62]. Also, it was recently shown that ERs can assume a type I integral membrane protein orientation in endothelial cells [63]. The predicted molecular model for this type of receptor comprises the hydrophobic Val-376–Val-392 region of ERα as the potential transmembrane domain, suggesting that multiple ER isoforms containing this hydrophobic sequence could conform membrane-spanning ERα [63]. Moreover, antibody-based imaging studies in living cells [60] and recent biotinylation experiments in astrocytes and neurons [22]–[24] also favor the idea of a mERα having an extracellular domain.

Several studies have shown that E2 tightly regulates the subcellular distribution of full-length ERα or other variants in different tissues [22]–[24], [60]. E2 causes trafficking of ERα to the plasma membrane within minutes in PC-12 cells, hypothalamic astrocytes and neurons, as well as pituitary cells [22]–[24], [60], [64]. Emerging evidence has identified a common mechanism for all gonadal steroid receptor trafficking to the plasma membrane. It involves post-translational S-palmitoylation on a conserved cysteine as part of a nine amino acid motif in the ligand binding domain of the receptor [65], [66], which in turn promotes its physical interaction with caveolin-1 facilitating the translocation of the receptor to caveolae rafts to the plasma membrane [18]. In the present study, we show that both full-length mERα and ∼39 kDa variant levels were higher at proestrus than at diestrus and that E2 induced a rapid increase in membrane levels of these mERα isoforms without modifying their intracellular expression. However, membrane levels of the ∼22 kDa variant of ERα, which may represent TERP-1 protein in a membrane location, remained unchanged during the estrous cycle or with E2 incubation. Thus, circulating levels of E2 could be involved in the increased expression of full-length mERα and mERα 39 kDa at proestrus, suggesting that these mERα variants may participate in the rapid apoptotic action of E2 in anterior pituitary cells. Our observations also suggest that E2 may increase trafficking of ERα variants to the plasma membrane rather than the synthesis of these proteins in anterior pituitary cells in the short term. Moreover, our results indicate that there is a ∼2 h window during which estradiol induces membrane trafficking of ERα, suggesting auto-regulation of membrane-initiated E2 signaling.

In conclusion, our results show that the rapid apoptotic action exerted by E2 in lactotropes depends on the presence of mERα and suggest that full-length ERα and/or a 39 kDa ERα variant are the likely candidates to mediate this action. Trafficking of ERα variants towards the plasma membrane may influence sensitivity of cells to acute estrogen action. E2 could thereby act through these membrane receptors to influence fine-tuning of anterior pituitary cell activity during the estrous cycle.

## References

[1] Shupnik MA (2002). Oestrogen receptors, receptor variants and oestrogen actions in the hypothalamic-pituitary axis.. J Neuroendocrinol.

[2] Lewis-Wambi JS, Jordan VC (2009). Estrogen regulation of apoptosis: how can one hormone stimulate and inhibit?. Breast Cancer Res.

[3] Jensen EV, Jacobson HI (1962). Basic guides to the mechanism of estrogen action.. Rec Prog Horm Res.

[4] McDevitt MA, Glidewell-Kenney C, Jimenez MA, Ahearn PC, Weiss J (2008). New insights into the classical and non-classical actions of estrogen: evidence from estrogen receptor knock-out and knock-in mice.. Mol Cell Endocrinol.

[5] Madak-Erdogan Z, Kieser KJ, Kim SH, Komm B, Katzenellenbogen JA (2008). Nuclear and extranuclear pathway inputs in the regulation of global gene expression by estrogen receptors.. Mol Endocrinol.

[6] Childs GV (2006). Physiology of Reproduction, Elsevier..

[7] Ben-Jonathan N, LaPensee CR, LaPensee EW (2008). What can we learn from rodents about prolactin in humans?. Endocr Rev.

[8] Sarkar DK (2006). Genesis of prolactinomas: studies using estrogen-treated animals.. Front Horm Res.

[9] Melmed S (2003). Mechanisms for pituitary tumorigenesis: the plastic pituitary.. J Clin Invest.

[10] Kawashima K, Yamakawa K, Takahashi W, Takizawa S, Yin P (2002). The estrogen-occupied estrogen receptor functions as a negative regulator to inhibit cell proliferation induced by insulin/IGF-1: a cell context-specific antimitogenic action of estradiol on rat lactotrophs in culture.. Endocrinology.

[11] Gutiérrez S, De Paul AL, Petiti JP, del Valle Sosa L, Palmeri CM (2008). Estradiol interacts with insulin through membrane receptors to induce an antimitogenic effect on lactotroph cells.. Steroids.

[12] Pisera D, Candolfi M, Navarra S, Ferraris J, Zaldivar V (2004). Estrogens sensitize anterior pituitary gland to apoptosis.. Am J Physiol Endocrinol Metab.

[13] Zárate S, Jaita G, Zaldivar V, Radl DB, Eijo G (2009). Estrogens exert a rapid apoptotic action in anterior pituitary cells.. Am J Physiol Endocrinol Metab.

[14] Zárate S, Zaldivar V, Jaita G, Magri L, Radl D (2010). The role of estrogens in anterior pituitary gland remodeling during the estrous cycle.. Front Horm Res.

[15] González M, Reyes R, Damas C, Alonso R, Bello AR (2008). Oestrogen receptor alpha and beta in female rat pituitary cells: an immunochemical study.. Gen Comp Endocrinol.

[16] Mitchner NA, Garlick C, Ben-Jonathan N (1998). Cellular distribution and gene regulation of estrogen receptors alpha and beta in the rat pituitary gland.. Endocrinology.

[17] Geffroy-Roisne S, Duval J, Thieulant ML (1992). Multiple forms of affinity-labeled estrogen receptors in rat distinct pituitary cells.. Endocrinology.

[18] Levin ER (2009). Plasma membrane estrogen receptors.. Trends Endocrinol Metab.

[19] Longo M, Brama M, Marino M, Bernardini S, Korach KS (2004). Interaction of estrogen receptor alpha with protein kinase C alpha and c-Src in osteoblasts during differentiation.. Bone.

[20] Denger S, Reid G, Kos M, Flouriot G, Parsch D (2001). ERalpha gene expression in human primary osteoblasts: evidence for the expression of two receptor proteins.. Mol Endocrinol.

[21] Li L, Haynes MP, Bender JR (2003). Plasma membrane localization and function of the estrogen receptor alpha variant (ER46) in human endothelial cells.. Proc Natl Acad Sci USA.

[22] Gorosito SV, Lorenzo AG, Cambiasso MJ (2008). Estrogen receptor alpha is expressed on the cell-surface of embryonic hypothalamic neurons.. Neuroscience.

[23] Bondar G, Kuo J, Hamid N, Micevych P (2009). Estradiol-induced estrogen receptor-alpha trafficking.. J Neurosci.

[24] Dominguez R, Micevych P (2010). Estradiol rapidly regulates membrane estrogen receptor alpha levels in hypothalamic neurons.. J Neurosci.

[25] Zivadinovic D, Watson CS (2005). Membrane estrogen receptor-alpha levels predict estrogen-induced ERK1/2 activation in MCF-7 cells.. Breast Cancer Res.

[26] Márquez DC, Pietras RJ (2001). Membrane-associated binding sites for estrogen contribute to growth regulation of human breast cancer cells.. Oncogene.

[27] Wang Z, Zhang X, Shen P, Loggie BW, Chang Y (2006). A variant of estrogen receptor-{alpha}, hER-{alpha}36: transduction of estrogen- and antiestrogen-dependent membrane-initiated mitogenic signaling.. Proc Natl Acad Sci USA.

[28] Shi L, Dong B, Li Z, Lu Y, Ouyang T (2009). Expression of ER-{alpha}36, a novel variant of estrogen receptor {alpha}, and resistance to tamoxifen treatment in breast cancer.. J Clin Oncol.

[29] Filardo EJ, Quinn JA, Bland KI, Frackelton AR (2000). Estrogen-induced activation of Erk-1 and Erk-2 requires the G protein-coupled receptor homolog, GPR30, and occurs via trans-activation of the epidermal growth factor receptor through release of HB-EGF.. Mol Endocrinol.

[30] Qiu J, Bosch MA, Tobias SC, Krust A, Graham SM (2006). A G-protein-coupled estrogen receptor is involved in hypothalamic control of energy homeostasis.. J Neurosci.

[31] Toran-Allerand CD, Guan X, MacLusky NJ, Horvath TL, Diano S (2002). ER-X: a novel, plasma membrane-associated, putative estrogen receptor that is regulated during development and after ischemic brain injury.. J Neurosci.

[32] Heberden C, Reine F, Grosse B, Henry C, Zagar Y (2006). Detection of a raft-located estrogen receptor-like protein distinct from ER alpha.. Int J Biochem Cell Biol.

[33] Muppidi J, Porter M, Siegel RM (2004). Measurement of apoptosis and other forms of cell death.. In: Current Protocols in Immunology. New York: Wiley..

[34] Gabriel L, Stevens Z, Melikian H (2009). Measuring Plasma Membrane Protein Endocytic Rates by Reversible Biotinylation. JoVE 34.. http://www.jove.com/index/Details.stp?ID=1669.

[35] Romero-Calvo I, Ocón B, Martínez-Moya P, Suárez MD, Zarzuelo A (2010). Reversible Ponceau staining as a loading control alternative to actin in Western blots.. Anal Biochem.

[36] Sun J, Huang YR, Harrington WR, Sheng S, Katzenellenbogen JA (2002). Antagonists selective for estrogen receptor alpha.. Endocrinology.

[37] Schaeffer M, Hodson DJ, Meunier AC, Lafont C, Birkenstock J (2011). Influence of estrogens on GH-cell network dynamics in females: a live in situ imaging approach.. Endocrinology.

[38] Bulayeva NN, Wozniak AL, Lash LL, Watson CS (2005). Mechanisms of membrane estrogen receptor-alpha-mediated rapid stimulation of Ca2+ levels and prolactin release in a pituitary cell line.. Am J Physiol Endocrinol Metab.

[39] Watson CS, Jeng YJ, Kochukov MY (2008). Nongenomic actions of estradiol compared with estrone and estriol in pituitary tumor cell signaling and proliferation.. FASEB J.

[40] Jeng YJ, Kochukov M, Watson CS (2010). Combinations of physiologic estrogens with xenoestrogens alter calcium and kinase responses, prolactin release, and membrane estrogen receptor trafficking in rat pituitary cells.. Environ Health.

[41] Sosa LD, Gutiérrez S, Petiti JP, Palmeri CM, Mascanfroni ID (2012). 17β-estradiol modulates the prolactin secretion induced by TRH through membrane estrogen receptors via PI3K/Akt in female rat anterior pituitary cell culture.. Am J Physiol Endocrinol Metab.

[42] Davis TL, Whitesell JD, Cantlon JD, Clay CM, Nett TM (2011). Does a nonclassical signaling mechanism underlie an increase of estradiol-mediated gonadotropin-releasing hormone receptor binding in ovine pituitary cells?. Biol Reprod.

[43] Watson CS, Campbell CH, Gametchu B (2002). The dynamic and elusive membrane estrogen receptor-alpha.. Steroids.

[44] Hart SA, Snyder MA, Smejkalova T, Woolley CS (2007). Estrogen mobilizes a subset of estrogen receptor-alpha-immunoreactive vesicles in inhibitory presynaptic boutons in hippocampal CA1.. J Neurosci.

[45] Yin P, Arita J (2002). Proestrus surge of Gonadotropin-releasing hormone secretion inhibits apoptosis of anterior pituitary cells in cycling female rats.. Neuroendocrinology.

[46] Zaldivar V, Magri ML, Zárate S, Jaita G, Eijo G (2009). Estradiol increases the Bax/Bcl-2 ratio and induces apoptosis in the anterior pituitary gland.. Neuroendocrinology.

[47] Mollard P, Hodson DJ, Lafont C, Rizzoti K, Drouin J (2012). A tridimensional view of pituitary development and function.. Trends Endocrinol Metab.

[48] Sanchez-Cardenas C, Fontanaud P, He Z, Lafont C, Meunier AC (2010). Pituitary growth hormone network responses are sexually dimorphic and regulated by gonadal steroids in adulthood.. Proc Natl Acad Sci USA.

[49] Castrique E, Fernandez-Fuente M, Le Tissier P, Herman A, Levy A (2010). Use of a prolactin-Cre/ROSA-YFP transgenic mouse provides no evidence for lactotroph transdifferentiation after weaning, or increase in lactotroph/somatotroph proportion in lactation.. J Endocrinol.

[50] Childs GV (2000). Growth hormone cells as co-gonadotropes: partners in the regulation of the reproductive system.. Trends Endocrinol Metab.

[51] Childs GV (2002). Development of gonadotropes may involve cyclic transdifferentiation of growth hormone cells.. Arch Physiol Biochem.

[52] Piroli GG, Grillo CA, Ferrini MG, Lux-Lantos V, De Nicola AF (1996). Antagonism by progesterone of diethylstilbestrol-induced pituitary tumorigenesis in Fischer 344 rats: effects on sex steroid receptors and tyrosine hydroxylase mRNA.. Neuroendocrinology.

[53] Caronti B, Palladini G, Bevilacqua MG, Petrangeli E, Fraioli B (1993). Effects of 17 beta-estradiol, progesterone and tamoxifen on in vitro proliferation of human pituitary adenomas: correlation with specific cellular receptors.. Tumour Biol.

[54] Brann DW, Rao IM, Mahesh VB (1988). Antagonism of estrogen-induced prolactin release by progesterone.. Biol Reprod.

[55] Cho BN, Suh YH, Yoon YD, Lee CC, Kim K (1993). Progesterone inhibits the estrogen-induced prolactin gene expression in the rat pituitary.. Mol Cell Endocrinol.

[56] Candolfi M, Jaita G, Zaldivar V, Zárate S, Ferrari L (2005). Progesterone antagonizes the permissive action of estradiol on tumor necrosis factor-alpha-induced apoptosis of anterior pituitary cells.. Endocrinology.

[57] Jaita G, Zárate S, Ferrari L, Radl D, Ferraris J (2011). Gonadal steroids modulate Fas-induced apoptosis of lactotropes and somatotropes.. Endocrine.

[58] Taylor SE, Martin-Hirsch PL, Martin FL (2010). Oestrogen receptor splice variants in the pathogenesis of disease.. Cancer Lett.

[59] Razandi M, Pedram A, Greene GL, Levin ER (1999). Cell membrane and nuclear estrogen receptors (ERs) originate from a single transcript: studies of ERalpha and ERbeta expressed in Chinese hamster ovary cells.. Mol Endocrinol.

[60] Gutiérrez S, Sosa LD, Petiti JP, Mukdsi JH, Mascanfroni ID (2012). 17β-Estradiol stimulates the translocation of endogenous estrogen receptor α at the plasma membrane of normal anterior pituitary cells.. Mol Cell Endocrinol.

[61] Christian HC, Morris JF (2002). Rapid actions of 17beta-oestradiol on a subset of lactotrophs in the rat pituitary.. J Physiol.

[62] Norfleet AM, Clarke CH, Gametchu B, Watson CS (2000). Antibodies to the estrogen receptor-alpha modulate rapid prolactin release from rat pituitary tumor cells through plasma membrane estrogen receptors.. FASEB J.

[63] Kim KH, Toomre D, Bender JR (2011). Splice isoform estrogen receptors as integral transmembrane proteins.. Mol Biol Cell.

[64] Alyea RA, Laurence SE, Kim SH, Katzenellenbogen BS, Katzenellenbogen JA (2008). The roles of membrane estrogen receptor subtypes in modulating dopamine transporters in PC-12 cells.. J Neurochem.

[65] Acconcia F, Ascenzi P, Bocedi A, Spisni E, Tomasi V (2004). Palmitoylation-dependent estrogen receptor alpha membrane localization: regulation by 17beta-estradiol.. Mol Biol Cell.

[66] Pedram A, Razandi M, Sainson RC, Kim JK, Hughes CC (2007). A conserved mechanism for steroid receptor translocation to the plasma membrane.. J Biol Chem.

